# Higher medical training in Rheumatology in the UK: any lessons worth learning?

**DOI:** 10.31138/mjr.28.4.171

**Published:** 2017-12-22

**Authors:** Andrew Hassell

**Affiliations:** Head of School of Medicine, Keele University, Staffordshire, United Kingdom, and Honorary Consultant Rheumatologist, Haywood Hospital, Stoke-on-Trent, United Kingdom

**Keywords:** United Kingdom, medical training, rheumatology training, postgraduate education

## Abstract

Across Europe, provision of high quality care for people with rheumatological conditions is at least partly dependent upon the rheumatologist, who generally plays a key role in making a diagnosis and in co-ordinating a patient’s care. In addition, in many countries the rheumatologist is pivotal in lobbying for services for patients with these disorders. It therefore follows that the training and accreditation of rheumatologists is important in ensuring high quality healthcare. In this commentary, I appraise some developments in the training of rheumatologists in the UK (training which has changed markedly over the past two decades). I do this as a means of promoting discussion.

## KEY ORGANISATIONS IN UK POSTGRADUATE MEDICAL EDUCATION

Health Education England^1^ is the key funder and overseer of postgraduate medical education in England (the devolved nations – Scotland, Wales and Northern Ireland - have separate bodies). Its work is delegated to 13 regional organisations (“Deaneries”).^2^ The Joint Royal Colleges Training Board^2^ defines the curriculum via the Rheumatology Specialist Advisory Committee. Training and the curriculum is regulated and approved by the General Medical Council (GMC). Satisfactory completion of higher medical training in rheumatology results in the awarding of a Certificate of Completion of Training (CCT), by the GMC (on the recommendation of the Specialist Advisory Committee), which allows the bearer to be entered on to the specialist register and thus to be eligible to take up a consultant rheumatologist post.

## SELECTION TO RHEUMATOLOGY TRAINING

Currently there is a national application process for trainees to enter rheumatology training. Applicants must have completed Foundation (2 years) and core medical (minimum 2 years) training and will usually hold MRCP. Application is made on a standardised online pro forma on which the applicant enters a hierarchy of preferred training programmes (these are based geographically in Deaneries). Applicants are invited to a central interview in which they are assessed in 3 separate “stations”, one focused on career questions, one on clinical competence and one on ethical understanding. Successful applicants are ranked and then matched with their preferred programme. There are strengths and weaknesses of this approach. It has the compelling virtues of being fair and transparent. Less attractive is that the national process can make it more challenging to develop the interest in rheumatology of trainees in a given geographical region if they then become subject to a national process. Also, the process can be bureaucratic, time consuming, and can result in the less popular regions struggling to recruit. Somehow recruitment issues appeared less of a problem when the selection process was regional.

## THE RHEUMATOLOGY CURRICULUM

There has been a formal curriculum in Rheumatology for several decades. Approved by the GMC, this is a formal description of the training required to be a rheumatologist in the UK.^3^ It includes a syllabus, a description of required skills, both generic and subject-specific, consideration of the nature of clinical attachments and responsibilities, and definition of the required assessments. It defines essential procedural skills (mainly joint and soft tissue injections). The curriculum was developed, and continues to be updated, by the Rheumatology Specialist Advisory Committee which includes heads of training programmes from across the country, trainee and lay representation. The curriculum is supported by an E-portfolio: an electronic repository of learning, reflection and assessments which is owned and maintained by the trainee, but over-seen by the trainee’s educational supervisor (a consultant with over-arching educational responsibility for the trainee whilst on a given placement). The E-portfolio forms a major part of the evidence by which a trainee is judged at the annual review of competence (below). Within the portfolio, most activities performed by the trainee are mapped to the curriculum.

Trainees undergo an “Annual Review of Competence”, undertaken by a training committee comprising rheumatology consultants from the region. This review determines whether the trainee has provided evidence of achievement of training goals and so can progress to the next year of training. The Rheumatology curriculum includes a decision aid^4^ to inform these annual review meetings, and contains over-arching descriptions of the level of practice a trainee should have achieved and the evidence that the trainee is expected to submit for each given year of training. Such evidence includes completed Supervised Learning Events, in which the supervisor observes the trainee dealing with patient, workplace based assessments such as Direct Observations of Procedural Skills (DOPS) assessments and multi-source feedback assessments, as well as evidence of engagement in audit, teaching and research. Prior to gaining the CCT, the trainee must have passed the Rheumatology Specialty Certificate Examination, a computer based multiple-choice (200 single best answers) paper delivered in bespoke test centres. It costs the trainee approximately 750 Euros to sit the examination.

We would contend that the curriculum is an essential and highly useful component of rheumatology training. Whilst the current version may have scope for improvement, it forms a clear definition of what is expected from the rheumatology trainee and from the training programme. Capturing such a curriculum can be invaluable in declaring what it is rheumatologists do and in catalysing discussions amongst stakeholders about training and education, both in the context of rheumatologists, but also of other related health professionals.

Regarding the evidence reviewed at the Annual Review of Competence, most would agree that this remains work in progress. The reports completed by the trainee’s supervising consultants do constitute valuable evidence if completed conscientiously. Similarly, multi-source feedback makes a useful contribution although many would contend that it has more to offer in terms of feedback.^6^ The other workplace based assessments have received mixed reviews.^7^ On the one hand, the concept of direct observation of the trainee performing consultations or carrying our practical procedures has high face validity. On the other hand, in medicine generally, there are reports of it degenerating into a “tick-box” exercise in which forms are sometimes completed with minimal or no direct observation of the trainee. Finally, there’s the Rheumatology Specialty Certificate examination, a relatively recent addition to the portfolio of evidence used in the UK. This approach has been adopted in the other physicians’ specialties too. Most would agree that, accepting the limitations of single best answer questions, this is useful as a formal assessment of knowledge and helps define the knowledge syllabus for trainees. Of course, it requires a lot of effort in terms of question writing and standard setting, but these activities are themselves useful continuing professional development for those involved.

## CREDENTIALING

In the UK, there is increasing interest in the concept of “credentialing”^7^ whereby the CCT holder may gain additional accreditation in sub-specialty areas of the discipline.^8^ Such accreditation might be gained while practising as a consultant. Areas of interest for such credentialing include metabolic bone disease, care of the adolescent with rheumatological disorders, and ultra-sound in rheumatology.

## QUALITY ASSURANCE OF TRAINING POSTS

A key aspect of training in Rheumatology in the UK is the quality assurance of training posts. The precise workings of this have changed over the years. Previously, programmes were formally visited by a quality assurance team comprising Specialty Advisory Committee members from elsewhere in the country. Now formal visits are rarer but there are still mechanisms in place to quality assure posts, not least an annual online survey of trainees in which they give quite detailed feedback about their posts. We’d suggest that the ending of the formal visits is something of a loss to the system. Not only did they constitute a very robust way of understanding and optimising programmes, they also were highly effective professional development for training programme directors, affording the opportunity of seeing other programmes and ideas in action.

One aspect of the quality assurance of rheumatology training is the experience and expertise in education of the trainee’s educational and clinical supervisors. This has gained significant attention in the UK over the last decade. As a minimum, supervisors must have attended some postgraduate training in supervision (“Training the Trainers” short courses). It is becoming increasingly common for some trainers to develop their interest further, attaining formal postgraduate qualifications in Medical Education, from Certificate to Masters programmes.

## CONCLUSION

Postgraduate training in rheumatology has developed considerably over the last 20 years. There have been key developments in terms of selection, supervision, assessment and quality assurance of training. **Table 1** includes a personal view on their strengths and weaknesses. Whilst some of these developments might not have clearly enhanced training, I would argue that others certainly have, including the development of portfolios and of competence assessment in this field. Many aspects of the approach have the potential to provide reassurance to patients and the public about the healthcare experts providing their care, surely a crucial issue. In an era of increasing geographic mobility (notwithstanding Brexit), the area of postgraduate medical training in rheumatology would make a fertile topic for a European symposium and collaboration.

**Table 1.** Some strengths and weaknesses of aspects of the current UK approach to higher medical training in Rheumatology.

	**Strengths**	**Weaknesses**
National selection process	Transparent and fair	Cumbersome. Weakens trainees’ ties to geographic regions
Explicit curriculum	Transparent. Good for trainees, trainers and the specialty	Requires regular review and updating
Knowledge examination	Clear expression of knowledge syllabus; fair; portable declaration of knowledge; writing questions is good CPD.	Question writing and standard setting is resource intensive. Expensive for the trainee
Workplace based assessment	Promote direct observation of the trainee; an excellent opportunity for focused feedback; multi-source feedback is robust in assessing professionalism	Sometimes becomes a “tick-box” exercise. Can be resource intensive. Not always valued
Award of Certificate of Completion of Training	A useful, portable, declaration of competence.	Significant costs and resource issues
Credentialing	Has the potential to accredit sub-speciality expertise. Useful to the holder, to employers and to patients	Yet to be successfully implemented
Quality assurance of training posts	Important for reassurance of the public and of trainees.	Can be resource intensive. If not carried out well can degenerate into tick-box exercise.
